# Large-scale multiferroic complex oxide epitaxy with magnetically switched polarization enabled by solution processing

**DOI:** 10.1093/nsr/nwz143

**Published:** 2019-10-08

**Authors:** Cong Liu, Feng An, Paria S M Gharavi, Qinwen Lu, Junkun Zha, Chao Chen, Liming Wang, Xiaozhi Zhan, Zedong Xu, Yuan Zhang, Ke Qu, Junxiang Yao, Yun Ou, Zhiming Zhao, Xiangli Zhong, Dongwen Zhang, Nagarajan Valanoor, Lang Chen, Tao Zhu, Deyang Chen, Xiaofang Zhai, Peng Gao, Tingting Jia, Shuhong Xie, Gaokuo Zhong, Jiangyu Li

**Affiliations:** 1 Shenzhen Key Laboratory of Nanobiomechanics, Shenzhen Institutes of Advanced Technology, Chinese Academy of Sciences, Shenzhen 518005, China; 2 School of Materials Science and Engineering, Xiangtan University, Xiangtan 411105, China; 3 School of Materials Science and Engineering, University of New South Wales, Sydney, NSW 2052, Australia; 4 Hefei National Laboratory for Physical Sciences at Microscale and Department of Chemical Physics, University of Science and Technology of China, Hefei 230026, China; 5 Institute for Advanced Materials and Guangdong Provincial Key Laboratory of Optical Information Materials and Technology, South China Academy of Advanced Optoelectronics, South China Normal University, Guangzhou 510006, China; 6 Dongguan Neutron Science Center, Dongguan 523803, China; 7 Department of Physics, Southern University of Science and Technology, Shenzhen 518005, China; 8 International Center for Quantum Materials and Electron Microscopy Laboratory, School of Physics, Peking University, Beijing 100871, China; 9 Hunan Provincial Key Laboratory of Health Maintenance for Mechanical Equipment, Hunan University of Science and Technology, Xiangtan 411201, China; 10 Department of Physics, College of Science, National University of Defense Technology, Changsha 410073, China; 11 Beijing National Laboratory for Condensed Matter Physics and Institute of Physics, Chinese Academy of Sciences, Beijing 100190, China; 12 Songshan Lake Materials Laboratory, Dongguan Neutron Science Center, Dongguan 523808, China

**Keywords:** complex oxide, solution method, epitaxy, multiferroic

## Abstract

Complex oxides with tunable structures have many fascinating properties, though high-quality complex oxide epitaxy with precisely controlled composition is still out of reach. Here we have successfully developed solution-based single-crystalline epitaxy for multiferroic (1-*x*)BiTi_(1-*y*)/2_Fe*_y_*Mg_(1-*y*)/2_O_3_–(*x*)CaTiO_3_ (BTFM–CTO) solid solution in large area, confirming its ferroelectricity at the atomic scale with strong spontaneous polarization. Careful compositional tuning leads to a bulk magnetization of 0.07 ± 0.035 μ_B_/Fe at room temperature, enabling magnetically induced polarization switching exhibiting a large magnetoelectric coefficient of 2.7–3.0 × 10^−7^ s/m. This work demonstrates the great potential of solution processing in large-scale complex oxide epitaxy and establishes novel room-temperature magnetoelectric coupling in epitaxial BTFM–CTO film, making it possible to explore a much wider space of composition, phase, and structure that can be easily scaled up for industrial applications.

## INTRODUCTION

Complex oxides with tunable compositions and structures have fascinating properties including high-temperature superconductivity [1], colossal magnetoresistance [2], superior piezoelectric effect [3], and room-temperature magnetoelectric coupling [4,5], and high-quality single-crystalline epitaxial films are essential for exploring their fundamental sciences and technological applications [6]. The composition of complex oxides, however, makes such epitaxial growth challenging via conventional physical vapor deposition (PVD) [7,8], and there is a strong desire to develop alternative strategies enabling complex oxide epitaxy, especially via solution processing. This is particularly important for room-temperature multiferroics that often requires sophisticated compositional engineering [9–12], e.g. to twist the antiferromagnetic ordering of bismuth ferrite (BFO) [13] into a ferromagnetic one, as recently demonstrated in the solid solution of (1-*x*)BiTi_(1-*y*)/2_Fe*_y_*Mg_(1-*y*)/2_O_3_–(*x*)CaTiO_3_ (BTFM–CTO) ceramics [11]. In particular, solid solution between BTFM and CTO has resulted in a morphotropic phase boundary (MPB) with enhanced piezoelectricity [14], while B-site doping is the primary strategy to optimize magnetic properties and magnetoelectric coupling [15], with Ti^4+^ and Mg^2+^ found to be effective for the stability of Bi-based perovskite [16]. High-quality epitaxy for oxides with compositions as complex as BTFM–CTO, however, remains out of reach, making it necessary to explore solution processing following earlier successes in sol–gel-based epitaxy for simpler oxides [17–19]. Here we develop sol–gel-based solution processing [20] for complex oxide epitaxy, with which we have successfully achieved large-scale single-crystalline epitaxial BTFM–CTO films with room-temperature multiferroicity and magnetically switched polarization that can be easily scaled up for industrial applications. We emphasize that high-quality epitaxy is essential for the interesting multiferroic properties revealed, which is rather difficult to achieve by the physical vapor-based processing that is more common in the field, and we are pleasantly surprised by the high-quality atomic structure observed, which is typically only seen in PVD-processed films.

**Figure 1.**
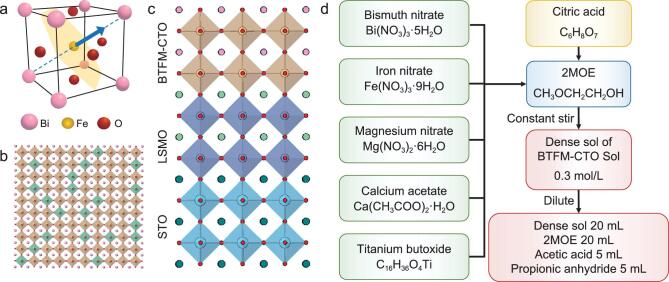
Crystalline lattice and solution processing of BTFM–CTO. Schematic diagrams of (a) (001)_pc_-oriented BFO crystal structure with ferroelectric polarization (blue arrow) and antiferromagnetic plane (shaded plane); (b) B-site doping and magnetic percolation of BTFM–CTO; (c) schematic epitaxial heterostructure of STO/LSMO/BTFM–CTO; (d) preparation of sol–gel precursor solutions.

We choose BTFM–CTO as our model system for its complex composition, whose baseline structure can be viewed as that of BFO [4] (Fig. 1a). The cycloidal spin structure of BFO can be disrupted by the solid solution of BTFM–CTO [11,21], resulting in magnetic percolation and bulk magnetization at *y* > 0.6 in BTFM–CTO ceramics [11] (Fig. 1b). In order to enable epitaxial growth of BTFM–CTO films, sol–gel-based two-step solution processing (Fig. 1d) has been developed using SrTiO_3_/La_0.7_Sr_0.3_MnO_3_ (STO/LSMO) (Fig. 1c) and Nb-doped SrTiO_3_ (NSTO) substrates, for which the misfit strain is evaluated to be −1.4% using the bulk lattice constants of 3.905 Å and 3.961 Å for STO and BTFM–CTO, respectively [11]. The processing is optimized at *x* = 0.14 near MPB and *y* = 0.8 for magnetic percolation, and the concentration of the solution is kept low to mitigate the evaporation rate during gelation, with propionic anhydride added to dehydrate the water in the solution and tunes its viscosity [8]. As a result, high-quality epitaxial films with typical sizes up to 20 × 20 mm^2^ (Fig. S1a in the Supplementary Information, SI) and atomic smooth surfaces have been obtained (Fig. S1b), exhibiting root-mean-square roughness as small as 115 pm in a scanning area of 3 × 3 μm^2^.

## RESULTS

### (001)-epitaxy of BTFM–CTO thin films

**Figure 2.**
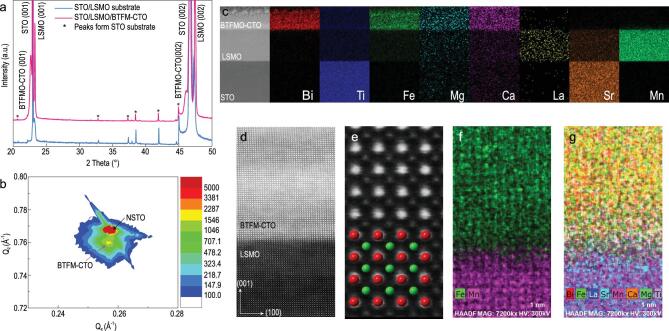
Epitaxial structure of BTFM–CTO. (a) XRD pattern of STO/LSMO/BTFM–CTO. (b) RSM of NSTO/BTFM–CTO at the (103) peak of NSTO. (c) STEM cross-sectional image and corresponding EDS element mapping. (d) Atomic-resolution HAADF image at the LSMO/BTFM–CTO interface viewed along [010]. (e) Magnified HAADF image of BTFM–CTO with crystal structure model with atom color in accordance with g. (f–g) Atomic-scale EDS mapping near the LSMO/BTFM–CTO interface.

We first examine the epitaxial growth of BTFM–CTO film via comprehensive structure characterizations. X-ray diffraction (XRD) ω-2θ patterns with 2θ from 20° to 50° of STO/LSMO/BTFM–CTO (Fig. 2a) and NSTO/BTFM–CTO (Fig. S2a) both demonstrate that BTFM–CTO films are epitaxially grown along the pseudocubic (001)_pc_ direction with no detectable secondary phase. The full width at half maxima (FWHM) of the rocking curve (Fig. S2b) scanned around the (001) diffraction peak is 0.0303^o^, indicating the high-quality epitaxy and excellent crystallinity. The reciprocal space map (RSM) of NSTO/BTFM–CTO measured around the (103) diffraction peak of STO (Fig. 2b) shows that the film possesses an identical in-plane lattice parameter to the substrate, demonstrated by the overlapping peak of the film and substrate along the *Q_x_* axis. Thus the film is coherently strained to the substrate with lattice constants of *a* = 3.905 Å, and *c* = 3.945 Å is determined from *Q_z_* as well. Low-magnification cross-sectional scanning transmission electron microscopy (STEM) of STO/LSMO/BTFM–CTO (Fig. 2c and Fig. S3a–c) was also carried out, showing a film thickness of 30.14 nm, while quantitative elemental analysis of STO/LSMO/BTFM–CTO based on energy-dispersive X-ray spectroscopy (EDS) mappings (Fig. 2c) reveals uniform distribution of all the elements. The atomically resolved high-angle annular dark field (HAADF) image (Fig. 2d) and selected area electron diffraction (SAED) pattern (Fig. S3d) of BTFM–CTO film on STO/LSMO substrate confirms the high-quality epitaxy at the atomic scale near the LSMO/BTFMO-CTO interface, with the viewing direction along [010]_pc_, and the magnified HAADF image of BTFM–CTO (Fig. 2e) matches well the lattice structure shown. Furthermore, high-resolution EDS maps for Fe, Mn and other elements (Fig. 2f–g) suggest that the interdiffusion of cations at the LSMO/BTFM–CTO interface is minor, limited to only a couple of unit cells. This set of data thus firmly establishes the high-quality epitaxial growth of BTFM–CTO single-crystalline films on STO/LSMO and NSTO substrates, quite remarkable for solution-based sol–gel processing.

### Ferroelectricity of epitaxial BTFM–CTO films

**Figure 3.**
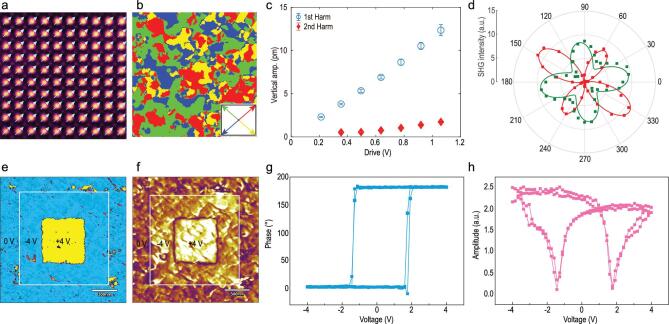
Ferroelectricity of STO/LSMO/BTFM–CTO. (a) Atomic-scale polarization vector of BTFM–CTO overlaid on a cross-sectional HAADF-STEM image. (b) Mapping of in-plane polarization, derived from two LPFM scans 90^o^ apart. (c) Comparison of first and second harmonic piezoresponses versus driving voltage. (d) Measured SHG signal represented as a polar diagram, where the green and red squares are the *p*- and *s*-polarized SHG signals, and the green and red lines are the corresponding fittings. (e–f) PFM phase and amplitude mappings of electrically poled domains. (g–h) PFM phase hysteresis and amplitude butterfly loops.

At the atomic scale, the polar order of BTFM–CTO is determined from the displacement of the B-site cation to the center of the surrounding A-site cations [22] using the atomically resolved HAADF image (Fig. 3a and Fig. S4), and the overlaid polarization vector is found to be uniformly distributed along the [011]_pc_ direction. The magnitude of this projected polarization is calculated to be 85–107 μC/cm^2^, comparable to 98 μC/cm^2^ reported for (001)_pc_-oriented BFO [23] and larger than ∼50 μC/cm^2^ measured for BTFM–CTO ceramics and polycrystalline film [11,24]. At the mesoscale, lateral piezoresponse force microscopy (LPFM) mappings before and after 90° sample rotation are carried out (Fig. S5) and then combined into in-plane polarization mapping (Fig. 3b), revealing an irregular domain pattern with a domain closure configuration. The corresponding vertical PFM (VPFM) mappings exhibit no phase contrast (Fig. S6), and thus the domain walls are determined to be mostly 71° type, though a small number of 109^o^ domain walls are also present [25]. Point-wise first and second harmonic vertical piezoresponses acquired under a series of excitation voltages (Fig. 3c) reveal that the electromechanical response is predominantly linear piezoelectric [26,27], consistent with the presence of strong polarization, which is also confirmed at the macroscopic scale by the polarization-dependent *p*- and *s*-polarized second harmonic generation (SHG) signals [28] shown in Fig. 3d. The polarization can be switched by an external electric field, as demonstrated by VPFM phase and amplitude mappings (Fig. 3e–f and Fig. S7) after box-in-box poling [29] by ±4 V as well as classical hysteresis and butterfly loops (Fig. 3g–h) acquired from point-wise switching spectroscopy [30]. This set of data thus establishes the ferroelectricity of epitaxial BTFM–CTO films, and we are currently working on fine-tuning their composition and processing to reduce leakage current for macroscopic hysteresis measurement.

### Magnetism of epitaxial NSTO/BTFM–CTO films

**Figure 4.**
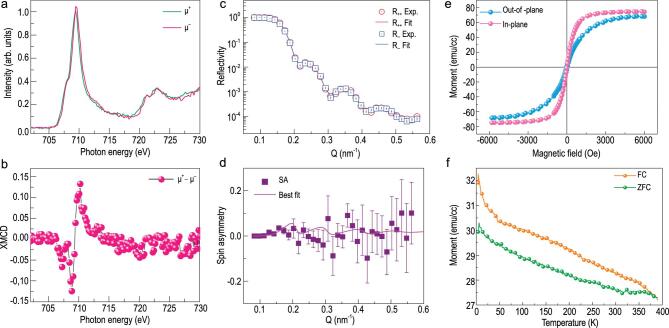
Magnetism of NSTO/BTFM–CTO. (a) XAS spectra at the Fe L_2,3_ edge under 4500 Oe. (b) XMCD calculated from a. (c) PNR with the spin-dependent neutron reflectivities *R*_++_ and *R*_−−_ at room temperature under a 7000 Oe magnetic field applied along the in-plane direction. (d) spin asymmetry (SA) calculated from c. (e) out-of-plane (blue) and in-plane (pink) magnetization curve versus applied magnetic field at room temperature. (f) ZFC (green) and FC (orange) temperature dependence of magnetization.

The X-ray absorption spectrum (XAS) of NSTO/BTFM–CTO is measured under a magnetic field of 4500 Oe with left- and right-hand circular lights, both of which exhibit the features corresponding to Fe^3+^ [31,32], with a left shoulder at ∼708 eV and a peak at ∼709.5 eV on the L_3_ edge (Fig. 4a). The X-ray magnetic circular dichroism (XMCD) signal is obtained by calculating the difference between the left- and right-hand XAS signals, showing strong dichroism on the L_3_ edge and non-observable dichroism on the L_2_ edge (Fig. 4b). From the spectra, a spin moment of ∼0.08 μ_B_/Fe and an orbital moment of ∼0.05 μ_B_/Fe are calculated, similar to 0.03 μ_B_/f.u. reported for Co-doped BFO [33]. We also use polarized neutron reflectometry (PNR) to examine NSTO/BTFM–CTO; this is a powerful tool for investigating the depth-resolved magnetization profile of thin films that is exclusively sensitive to the long-range order and thus can eliminate the signals resulting from magnetic contamination or cluster [34]. The non-spin-flip specular reflectivities of polarized neutrons (*R*_++_ and *R*_−−_, Fig. 4c) are dependent on the sample magnetic and nuclear scattering length density denoted as mSLD and nSLD (Fig. S8), from which the spin asymmetry $\mathrm{SA}(Q)=\frac{R_{++}{-}{R}_{--}}{R_{++}+{R}_{--}}$ as a function of wave vector transfer *Q* can be calculated (Fig. 4d). Note that mSLD is directly proportional to the magnetization of the sample since it is much smaller than nSLD, and the magnetization trends can be readily extracted by examining the magnitude of the SA features [34]. The difference between *R*_++_ and *R*_−−_ is rather small, and the best fit yields a magnetization of 0.05 ± 0.024 μ_B_/f.u., corresponding to 0.07 ± 0.035 μ_B_/Fe. This is consistent with the magnetization determined from XMCD and is larger than 0.0097 μ_B_/Fe reported for bulk BTFM–CTO ceramics [9]. The magnetic hysteresis (M-H) loop measured at room temperature demonstrates weak ferromagnetism with a saturated magnetic moment of ∼72 emu/cm^3^ at 6000 Oe (Fig. 4e), and remnant magnetization out-of-plane is estimated to be ∼3 emu/cm^3^ (Fig. S9). The saturation magnetization is larger than that estimated from XMCD and PNR, suggesting the existence of oxygen vacancies and impurities that cannot be detected by PNR [35,36]. It may also arise from NSTO substrate as already reported in the literature [37]. Splitting of zero-field-cooling (ZFC) and field-cooling (FC) magnetization-temperature (M-T) curves under a detecting field of 200 Oe occurs at ∼370 K (Fig. 4f), consistent with the value reported in BTFM–CTO ceramics [11]. This set of data thus establishes room-temperature bulk magnetization in epitaxial BTFM–CTO film.

### Magnetically switched ferroelectric polarization

**Figure 5.**
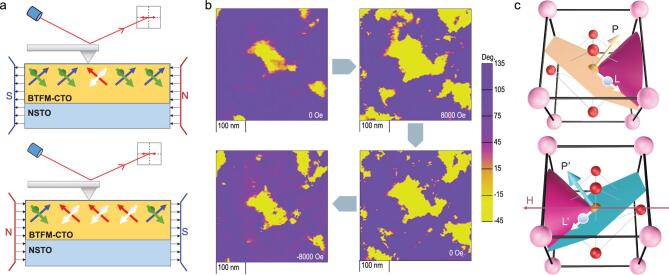
Magnetoelectric coupling of BTFM–CTO. (a) Schematic experimental set-up for polarization switching under an in-plane magnetic field; blue and red arrows indicate polarization while green and white arrows indicate net magnetization. (b) LPFM domains expand, maintain and shrink under different in-plane magnetic fields. (c) Proposed polarization switching pathway under the applied magnetic field; *P* and *P* ^′^ denote polarization before and after switching, while *L* and *L*^′^ represent the corresponding magnetization.

The ferromagnetism in epitaxial BTFM–CTO, in combination with its coupling with polarization, raises an exciting prospect of switching polarization by an external magnetic field at room temperature in a single-phase thin film, and thus we examine LPFM domain patterns of NSTO/BTFM–CTO film under the influence of opposite in-plane magnetic fields [10,38]. In-plane domains with 180° phase contrast are evident in the original LPFM mapping in the absence of any magnetic field (Fig. 5a) and, upon the application of +8000 Oe field, yellow domains expand at the expense of purple domains (Fig. 5b), while the VPFM contrast is intact (Fig. S10). The fraction of the switched polarization is estimated to be 37%, and the magnetoelectric coefficient is calculated to be $\frac{\boldsymbol{\Delta P}}{\boldsymbol{\Delta H}}=$ 2.7–3.0 × 10^−7^ s/m, in comparison to 1.3 × 10^−7^ s/m estimated for a solid solution of lead zirconium titanate (PZT) and lead iron tantalate (PFT) [10]. When this magnetic field is

removed, the ferroelectric domains are maintained, suggesting that the magnetically switched polarizations are non-volatile. A magnetic field of −8000 Oe opposite to the original field then switches the ferroelectric domain back to the original configuration, and similar behavior is also observed in a larger region (Fig. S11) along with corresponding mappings of topography and LPFM amplitude, as well as in different samples (Fig. S12). Throughout the processes, the topography is unchanged (Fig. S11a–d), and thus PFM measurement under high AC excitation is unlikely to be affected by the quasi-static magnetic field. Note that polarization is perpendicular to the easy magnetic plane (Fig. 5c), and thus under an external magnetic field opposite to the magnetization, the magnetic moment rotates around the applied magnetic field, forming a cone with the magnetic field as the axis. This eventually results in the flipping of the magnetization easy plane to reduce the angle between the magnetic field and moment, leading to simultaneously switched in-plane polarization as observed in Fig. 5b, though understanding the exact mechanisms requires further investigation. Additional coupling between polarization and magnetic field is also possible, and similar observations have also been reported in other materials [10,39].

## DISCUSSION

Bismuth ferrite (BFO) is the most widely investigated multiferroic oxide [12], and the ability to fabricate high-quality single-crystalline epitaxial BFO on a variety of substrates, most commonly by pulse laser deposition (PLD), makes it possible to tune its structure and properties via strain engineering. This contributes to the great versatility and wide popularity of BFO and has resulted in many intriguing properties [4,5,22,23,25,32]. The antiferromagnetic ordering of bismuth ferrite, however, limits its bulk magnetization [5,13], and it is quite challenging to manipulate its polarization via magnetic means. Here, by developing high-quality epitaxy for BTFM–CTO solid solution with precisely controlled and conveniently tuned compositions via the sol–gel method, we achieve rare bulk magnetism in epitaxial BTFM–CTO films, enabling polarization switching by magnetic field. We believe this opens many exciting opportunities, e.g. enabling exploration of the otherwise inaccessible space of phase and structure by continuously tuning the complex compositions in combination with strain engineering. Many exciting applications can be envisioned as well, e.g. ferroelectric field effect transistors (FeFET) that can be magnetically switched, though out-of-plane magnetic switching is preferred to realize such devices. We are currently working on these exciting prospects.

## CONCLUSIONS

In conclusion, we have successfully developed sol–gel-based complex oxide epitaxy for multiferroic BTFM–CTO solid solution in large area, enabling convenient compositional tuning in combination with strain engineering for magnetically switched polarization. The process is simple, fast, cost-effective, and can be easily scaled up for industrial applications, making it possible to explore a much wider space of composition, phase, and structure of complex oxides.

## Supplementary Material

nwz143_Supplemental_File

## Data Availability

The data that support the findings of this study are available from the corresponding author upon reasonable request.
