# Supplementary material for: Large-scale multiferroic complex oxide epitaxy with magnetically switched polarization enabled by solution processing
Source: Natl Sci Rev. 2019 Oct 8;7(1):84–91. doi: 10.1093/nsr/nwz143 (PMC8289034; doi:10.1093/nsr/nwz143)
Supplement: nwz143_Supplemental_File [file nwz143_supplemental_file.docx]

**Supplementary Information for**

**Large-scale Multiferroic Complex Oxide Epitaxy with Magnetically Switched Polarization Enabled by Solution Processing**

Cong Liu^1†^, Feng An^1,2†^, Paria S.M. Gharavi^3^, Qinwen Lu^4^, Junkun Zha^4^, Chao Chen^5^, Liming Wang^6^, Xiaozhi Zhan^6^, Zedong Xu^7^, Yuan Zhang^2^, Ke Qu^1,8^, Junxiang Yao^1^, Yun Ou^1,9^, Zhiming Zhao^2^, Xiangli Zhong^2^, Dongwen Zhang^10^, Nagarajan Valanoor^3^, Lang Chen^7^, Tao Zhu^6,11,12^, Deyang Chen^5^, Xiaofang Zhai^4^, Peng Gao^8^, Tingting Jia^1*^, Shuhong Xie^2*^, Gaokuo Zhong^1*^, Jiangyu Li^1*^

^1^ Shenzhen Key Laboratory of Nanobiomechanics, Shenzhen Institutes of Advanced Technology, Chinese Academy of Sciences, Shenzhen, 518005, China.

^2^ School of Materials Science and Engineering, Xiangtan University, Xiangtan, 411105, China.

^3^ School of Materials Science and Engineering, University of New South Wales, Sydney, NSW 2052, Australia.

^4^ Hefei National Laboratory for Physical Sciences at Microscale and Department of Chemical Physics, University of Science and Technology of China, Hefei, 230026, China.

^5^ Institute for Advanced Materials and Guangdong Provincial Key Laboratory of Optical Information Materials and Technology, South China Academy of Advanced Optoelectronics, South China Normal University, Guangzhou, 510006, China.

^6^ Dongguan Neutron Science Center, Dongguan, 523803, China.

^7^ Department of Physics, Southern University of Science and Technology, Shenzhen, 518005, China.

^8^ International Center for Quantum Materials and Electron Microscopy Laboratory, School of Physics, Peking University, Beijing, 100871, China.

^9^ Hunan Provincial Key Laboratory of Health Maintenance for Mechanical Equipment, Hunan University of Science and Technology, Xiangtan, 411105, China.

^10^ Department of Physics, College of Science, National University of Defense Technology, Changsha, 410073, China.

^11^ Beijing National Laboratory for Condensed Matter Physics and Institute of Physics, Chinese Academy of Sciences, Beijing, 100190, China.

^12^ Songshan Lake Materials Laboratory, Dongguan Neutron Science Center, Dongguan, 523808, China.

^†^ Equally contributed to this work.

^*^ Corresponding authors. E-mails: [jy.li1@siat.ac.cn](mailto:jy.li1@siat.ac.cn); [gk.zhong@siat.ac.cn](mailto:gk.zhong@siat.ac.cn); [shxie@xtu.edu.cn](mailto:shxie@xtu.edu.cn); [tt.jia@siat.ac.cn](mailto:tt.jia@siat.ac.cn)

**Methods**

**BTFM-CTO Solution preparation.** Solution of 0.86BiTi_3/8_Fe_2/8_Mg_3/8_O_3_–0.14CaTiO_3_ was prepared by two steps. First, sufficiently dissolving the starting materials of bismuth nitrate (Bi(NO_3_)_3_·5H_2_O, ACS 98%, Aladdin), iron nitrate (Fe(NO_3_)_3_·9H_2_O, ACS 98.5%, Aladdin), magnesium nitrate (99%, Sigma Aldrich), calcium acetate (99%, Aladdin), and butyl titanate (99%, Aladdin) one by one in 2-methoxyethanol (2-MOE, ACS >99.5%, Aladdin) solvent, with 5% excess bismuth nitrate added to compensate the evaporation of bismuth during the annealing process. Then citric acid (ACS >99.7%, Aladdin) was added into the precursor solution as chelation agent. Keep the solution in constant stirring for 12 hours to obtain the 0.3 mol/L dense solution. In the second step, 10 mL propionic anhydride (GC >98.5%, Aladdin) was added into the mixture of 20 mL dense solution and 20 mL 2-MOE to dehydrate the water of crystallization from the metal salts, and then stirred for another 12 hours and aged for 72 hours to obtain the diluted solution.

**Spin-coating and annealing process.** The substrates were first cleaned by oxygen plasma for 100 seconds. The solution was then spin-coated onto the substrates sequentially at 600 r/min for 7 seconds and 5000 r/min for 15 seconds. After deposition of each layer, the samples were transferred onto the heating stage with temperature of 180 °C to accelerate the condensation reaction and then pyrolyzed at 380 °C for 15 minutes to remove the organic frameworks. After three layers of spin-coating and pyrolysis process, the samples were annealed at 800 °C for 15 minutes in a rapid thermal processing furnace under oxygen atmosphere, and a cooling rate of 0.25 °C/s is found to be stable for high quality crystallization.

**LSMO buffer layer growth method.** La_0.7_Sr_0.3_MnO_3_ (LSMO) buffer layer on SrTiO_3_ substrate was deposited by pulsed laser deposition (PLD) at 700 °C in an oxygen ambient of 100 mTorr at a growth rate of ~0.7 Å/sec, with 9600 pulses. The films were cooled at 10 °C/min to the room temperature in an oxygen ambient of 1 atmos.

**X-ray diffraction and reciprocal space mapping.** The phase composition was determined by the powder X-ray diffraction (XRD, Bruker D8 Focus X-ray diffraction, Germany) using the Cu K_α_ radiation (λ=1.5406 Å). The reciprocal space mapping of the thin film was characterized by a 4-circle diffractometer.

**Rocking curve.** Rocking curve scan around the BTFM-CTO (001) diffraction was examined by X-ray diffraction (PANalytical X’Pert PRO). The step size and time per step during scanning are 0.002 degrees and 0.5 seconds, respectively. The FWHM of the peak is 0.03034 degrees, which indicated the high-quality epitaxy and excellent crystallinity.

**Scanning transmission electron microscopy.** Cross-sectional TEM sample of approximately 40 nm thickness was prepared by focused ion beam (FIB) using lift-out technique. High-angle annular dark field (HAADF)-STEM, bright-field (BF)-STEM imaging, and selective area electron diffraction (SAED) patterns were obtained by a Cs-corrected transmission electron microscope (JEOL JEM-ARM200F) operated at 200 kV). High-resolution HAADF STEM images were acquired at an aberration-corrected TEM (FEI Titan Cubed Themis G2 60-300) operated at 300 kV equipped with an XFEG gun and Bruker Super-X EDX detectors. STEM images were acquired with a beam current of 0.05-0.1 nA, a convergence semi-angle of 25 mrad, and a collection semi-angle snap in the range of 53-260 mrad. The STEM-EDX mapping was obtained with a beam current of 0.2-0.3 nA and counts ranging from 2 k cps to 8 k cps for ~5 minutes.

**Piezoelectric force microscopy measurement.** The lateral and vertical piezoelectric force microscopy (LPFM and VPFM) were performed with an Asylum Research Cypher-ES atomic force microscope (AFM) using dual ac resonance tracking PFM (DART-PFM) by a conductive probe with a spring constant of 2 N/m (FMG-01-Pt) in contact mode with scan rate of 0.5 Hz per line. SSPFM measurement is adopted to illustrate polarization switching by applying a DC voltage up to 4 V, and detected by an ac voltage of 0.5 V at each DC step. Variable field module (VFM) is used to apply an adjustable in-plane magnetic field under AFM.

**Second harmonic generation measurement.** The SHG measurements were achieved in reflection configuration with an incident angle of 45°. The 800 nm laser (laser amplifier system, 150 fs, 10 nJ, 76 MHz) are used as incident fundamental beam (electric field *E_ω_*). The sample are installed along STO [100] direction. The polarization-dependent measurements were obtained by rotating the polarization angle of the incident fundamental beam through a half-wave plate, where the 0° or 180° corresponds to *p* polarization (*p*_in_), and 90° or 270° corresponds to *s* polarization (*s*_in_), respectively. The *p*-polarized and *s*-polarized SHG signals are analyzed by Glan prisms.

**X-ray magnetic circular dichroism measurement.** The XAS and XMCD measurements were performed on beamline BL08U1A at the Shanghai Synchrotron Radiation Facility. The measurements were performed at room temperature and under vacuum pressure of less than 1×10^−6^ Pa. The XAS data measured using the left- and right-hand circular light were recorded in total electron yield mode (TEY) and normalized by the incident photon flux. The XMCD signal is then calculated as the difference of the left-hand and right-hand XAS signals. The samples were measured with alternating left-polarized (μ+) and right-polarized (μ-) photons in an applied field of 4500 Oe. Both the X-ray beam and the magnetic field were perpendicular to the sample surface.

**Polarized neutron reflectivity measurement.** Polarized neutron reflectivity (PNR) measurements were carried out at the multipurpose reflectometer (MR) in China Spallation Neutron Source (CSNS) [1]. PNR curves were recorded at a magnetic field of 7000 Oe at room temperature, far in excess of the necessary field to saturate the sample. The samples had dimensions of 20×20 mm^2^ on a 0.5 mm thick STO (100) substrate. PNR data were fitted using the default layer model in Genx software package [2]. In the model, the volume ratio of BTFM and CTO was set as 0.87 to 0.13, while the literate values of scattering length and density of the composite were calculated from NIST website. The NSTO substrate is treated as slabs with uniform nSLD and zero mSLD. The best fit gives a Factor of Merit (FOM) of 0.0038.

**Superconducting quantum interference device measurement.** The M-H (magnetization versus magnetic field) and the M-T (magnetization versus temperature) were measured by a Quantum Design superconducting quantum interference device (SQUID) measurement system (MPMS-7) in the temperature range 10-390 K with the magnetic field (H) applied along the (100) direction of the substrate after cooling down to 10 K in a magnetic field of 6000 Oe and 0 Oe, respectively.

**LPFM measurement under applied magnetic filed.** The magnetoelectric measurements was operated on an MFP-3D-BIO AFM equipped with the external in-plane variable field module (VFM3, Asylum Research). The sample was placed on VFM3 stage to realize *in-situ* observation of the ferroelectric domain evolution under in-plane different magnetic field.


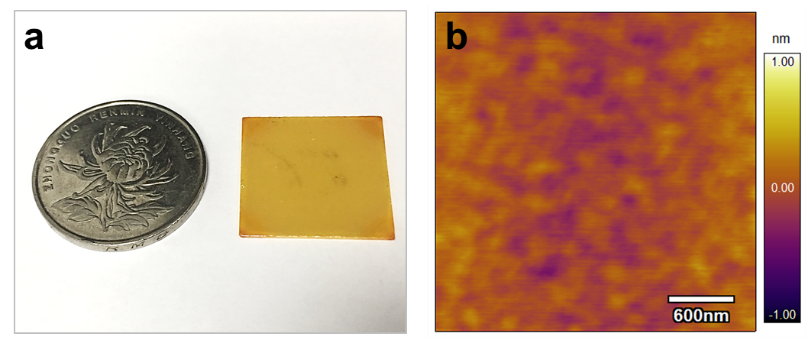


**Figure S1. (a)** Optic photo of Sol-Gel derived 20×20 mm^2^ high quality thin film on STO (100) substrate. **(b)** AFM topography mapping of an ultra-flat NSTO/BTFM-CTO thin film.


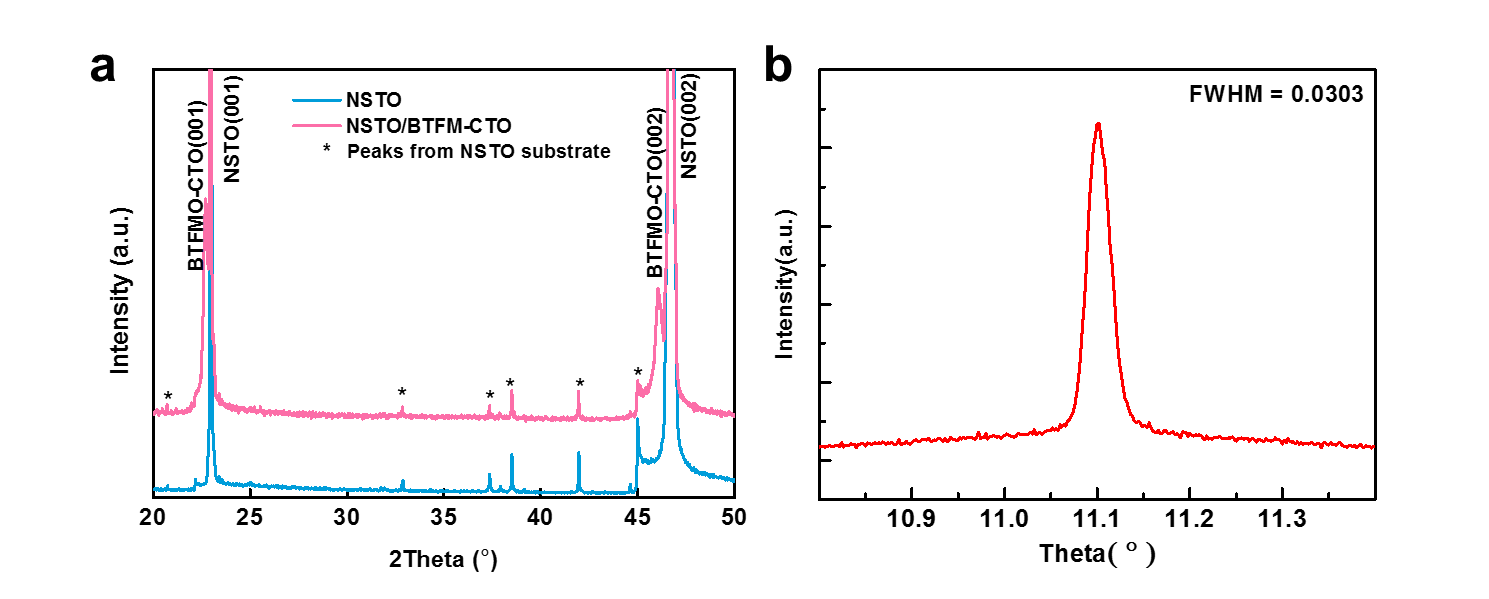


**Figure S2. (a)** XRD pattern of sample NSTO/BTFM-CTO (pink) and NSTO substrate (blue). **(b)** Rocking curve of BTFM-CTO (001) peak.


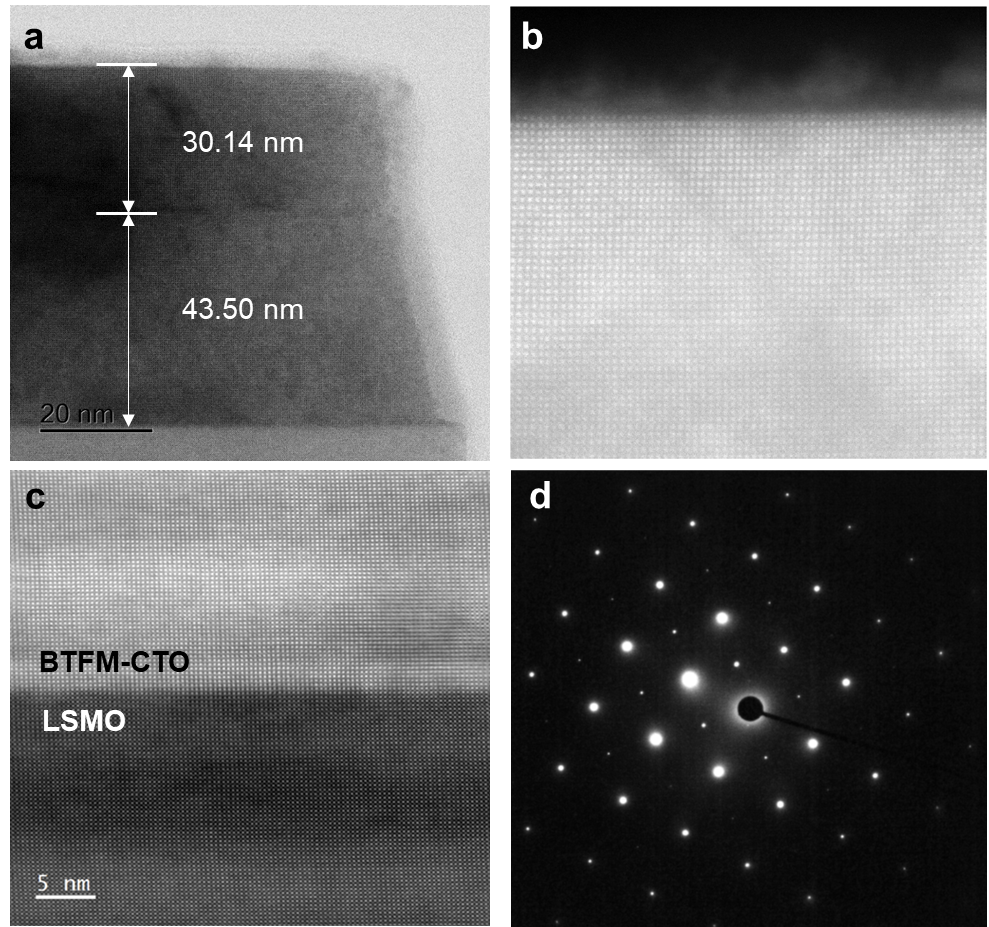


**Figure S3. (a)** Low magnification cross-sectional image of the sample STO/LSMO/BTFM-CTO demonstrates the thin film thickness of 30.14 nm. **(b)** Partial enlarged photo of the surface area illustrates an atomic level flatness of the sample surface. **(c)** Partial enlarged area at the BTFM-CTO/LSMO interface clearly shows an orderly epitaxial growth of BTFM-CTO thin film. **(d)** Selected arear electron diffraction pattern of BTFM-CTO/LSMO area reveals orderly epitaxial growth with no polycrystal-characteristic diffraction rings.


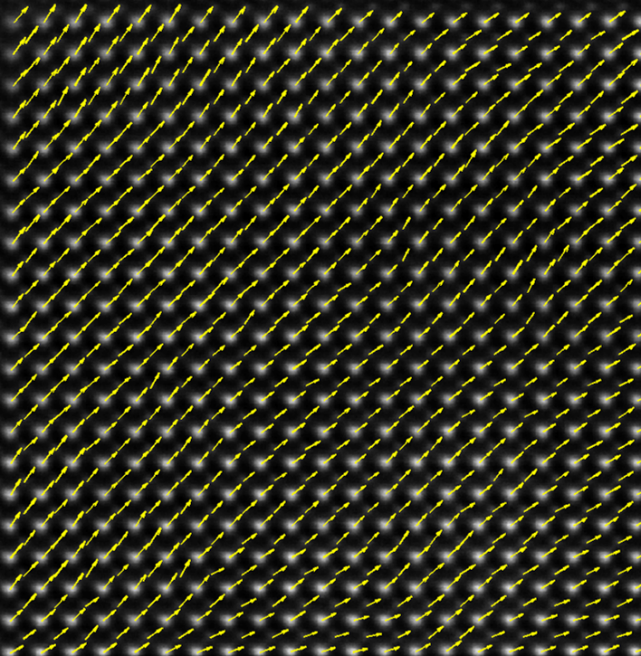


**Figure S4.** ABSF filtered image of a cross-sectional HAADF-STEM in larger region.

**Figure S5.** LPFM morphology, amplitude, phase and binarized phase mapping at the same area of STO/LSMO/BTFM-CTO before **(a-d)** and after **(e-h)** sample rotation of 90° .

**Figure S6.** VPFM **(a)** morphology, **(b)** amplitude and **(c)** phase mapping at the same area as **Fig. S5**, demonstrating single-direction vertical polarization of the film.

**Figure S7.** Bias-voltage pattern and VPFM morphology, amplitude and phase mapping of **(a-d)** STO/LSMO/BTFM-CTO in comparison with that of **(e-h)** NSTO/BTFM-CTO. Opposite as-grown vertical polarization directions were due to the self-polling effect by the bottom electrode potential.


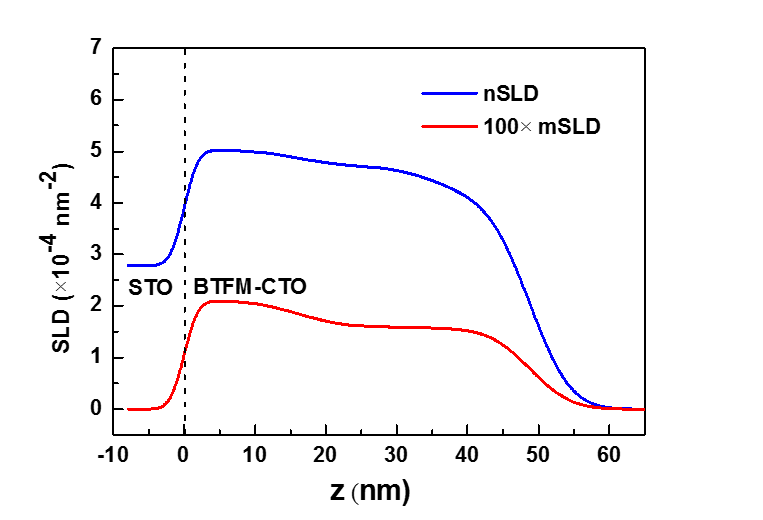


**Figure S8.** Nuclear scattering length density of STO substrate and magnetic scattering length density of BTFM-CTO thin film measured by PNR.

**Figure S9.** Center enlarge of M-H loop in **Fig. 4e**.


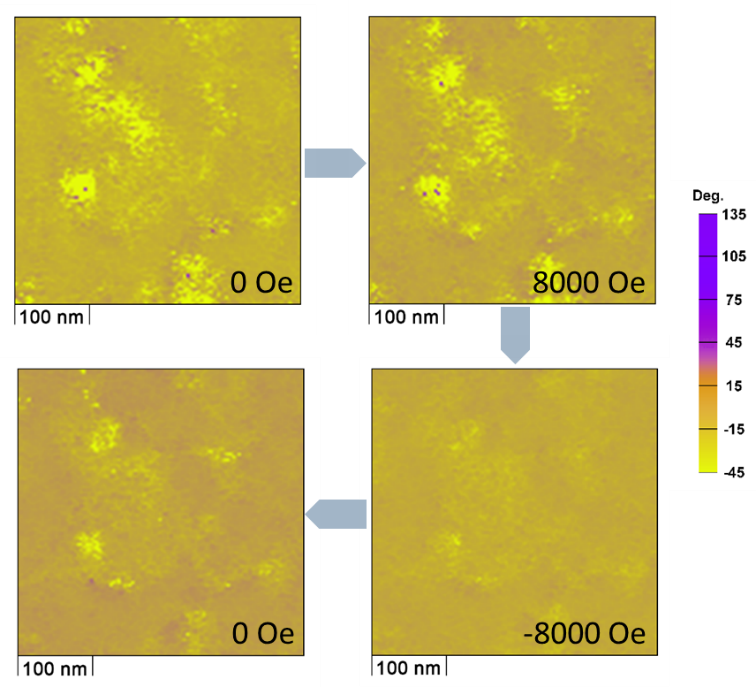


**Figure S10.** VPFM phase mapping under different in-plane magnetic field of the same area as **Fig. 5b**.


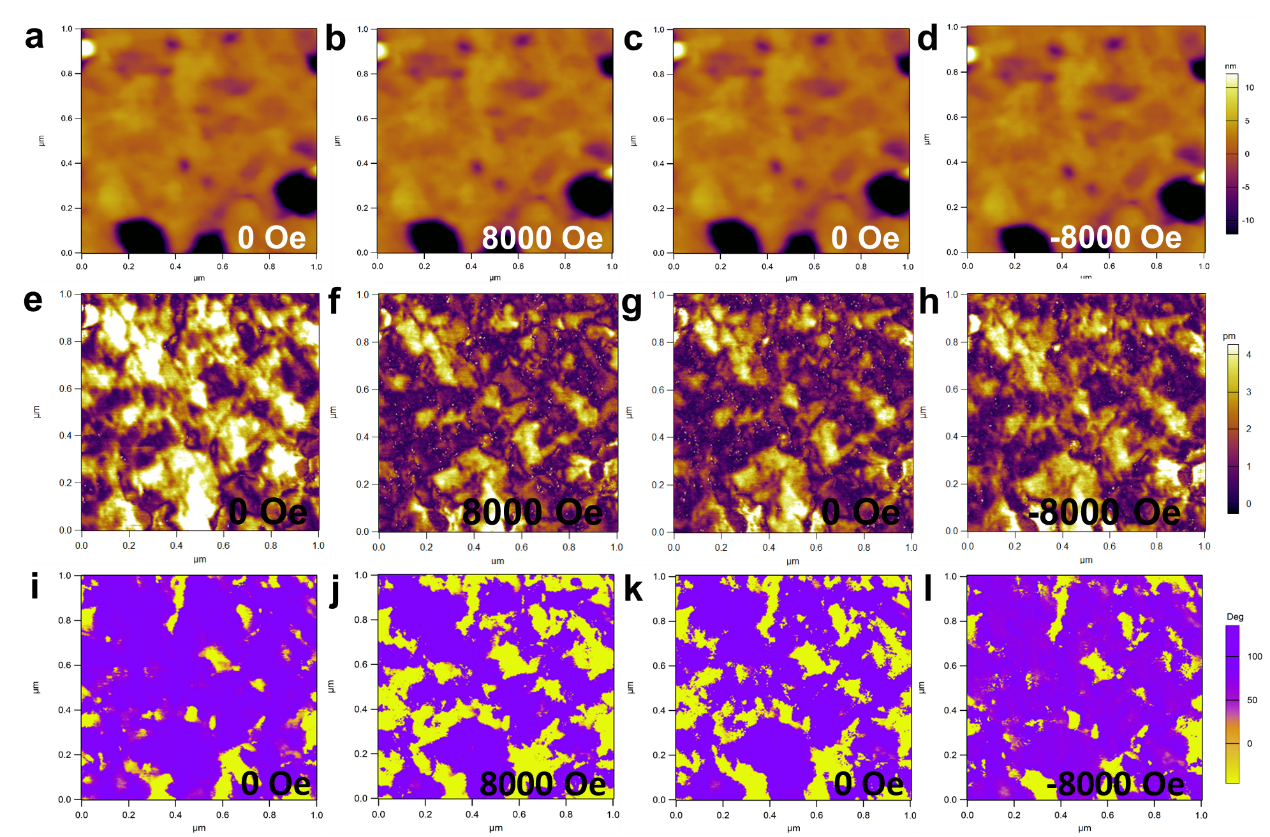


**Figure S11. (a-d)** Larger region morphology, **(e-h)** LPFM Amplitude and **(i-l)** phase mapping of NSTO/BTFM-CTO under different external in-plane magnetic field in accordance with **Fig. 5b**.


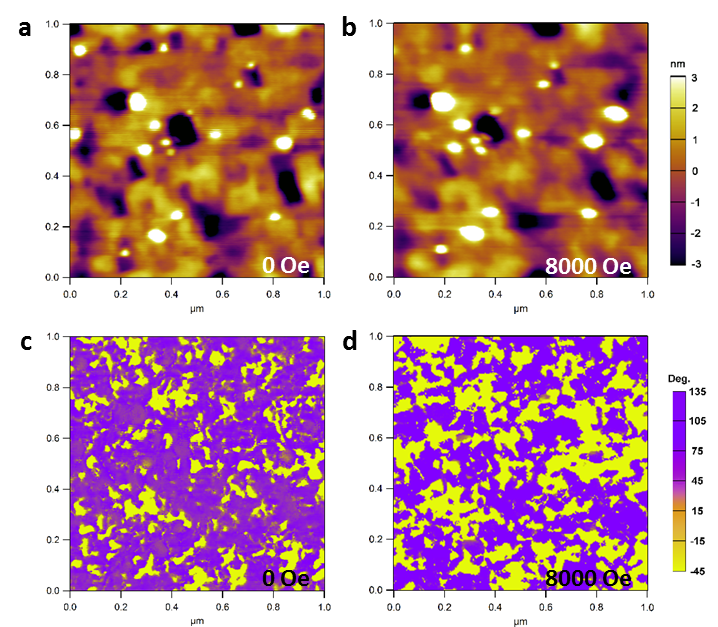


**Figure S12.** **(a-b)** Morphology and **(c-d)** LPFM phase mapping of another NSTO/BTFM-CTO sample under different external in-plane magnetic field.

**REFERENCES**

1. Zhu T, Zhan X and Xiao S *et al.* MR: The multipurpose reflectometer at CSNS. *Neutron News* 2018; **29**: 11–13.

2. Björck M and Andersson G. GenX: An extensible X-ray reflectivity refinement program utilizing differential evolution. *J Appl Crystallogr* 2007; **40**: 1174–1178.
